# Evolutionary diversification of taiwanioid conifers: evidence from a new Upper Cretaceous seed cone from Hokkaido, Japan

**DOI:** 10.1007/s10265-020-01214-y

**Published:** 2020-07-19

**Authors:** Ruth A. Stockey, Harufumi Nishida, Gar W. Rothwell

**Affiliations:** 1 Department of Botany and Plant Pathology, Oregon State University, Corvallis, OR 97331 USA; 2 Department of Biological Sciences, Faculty of Science and Engineering, Chuo University, 1-13-27 Kasuga, Bunkyo, Tokyo, 112-8551 Japan; 3 Graduate School of Biological Science, University of Tokyo, Tokyo, Japan; 4 Department of Environmental and Plant Biology, Ohio University, Athens, OH 45701 USA

**Keywords:** Anatomy, Conifer seed cone, Cupressaceae, Hokkaido, Late Cretaceous, Taiwanioid diversity

## Abstract

A single cylindrical seed cone 2 cm long, 1.1 cm wide has been found preserved in a calcium carbonate marine concretion from the Hakobuchi Formation (late Campanian-early Maastrichtian) of Hobetsu, Hokkaido, Japan. The cone, attached to a bent peduncle lacking leaves, has helically arranged bract/scale complexes that arise at right angles from the cone axis in the middle of the cone. The cone axis, ca. 1 mm wide, has a broad cylinder of secondary vascular tissue, and lacks a continuous resin canal system. Bract-scale complexes are laminar, cordate-orbiculate, and upturned distally, consisting primarily of bract tissue with no visible scale tip. The vascular trace to the bract/scale complex originates as a rod that divides laterally into several traces at the level of seed attachment. A single resin canal originates at the base of the bract-scale complex abaxial to the vascular strand, but more distally there are up to ca. 15 large resin canals that form a single row. Two to three inverted winged seeds are attached adaxially near the cone periphery. Cone structure and vascularization are most similar to those in the Cupressaceae, Subfamily Taiwanioideae, differing from living *Taiwania cryptomerioides* by having up to three seeds/scale rather than two, an abruptly upturned bract tip, in details of bract/scale vasculature, and a cone peduncle lacking leaves. This cone is described as *Mukawastrobus satoi* Stockey, Nishida and Rothwell. Together with previously described Early to Late Cretaceous taiwanioid seed cones from Mongolia and Hokkaido the new species demonstrates that the taxonomically diagnostic characters of such conifers are as subtle as those of Cretaceous and Cenozoic sequoioid Cupressaceae. This realization emphasizes that evolutionary diversification and turnover among taiwanioid conifers during the Cretaceous and Paleogene are probably far greater than currently recognized.

## Introduction

Although there are reports of more ancient fossils, empirical evidence from the paleontological record documents that the conifer family Cupressaceae originated no later than the mid-Jurassic (Escapa et al. 2008; Rothwell et al. 2011; Stockey et al. 2005). Stem group Cupressaceae flourished during the Late Jurassic and Early Cretaceous (e.g., *Austrohamia* Escapa, Cúneo et Axsmith spp., *Sewardiodendron* spp., *Elatides* spp.), and several crown group subfamilies evolved by the late Jurassic (Rothwell et al. 2011; Spencer et al. 2015). These subfamilies include Cunninghamioideae (i.e., *Hughmillerites* Rothwell et al. 2011), Taiwanioideae, Athrotaxoideae, Sequoioideae, and Taxodioideae (Herrera et al. 2017; Rothwell and Stockey 2018; Stockey et al. 2005, 2018). Both the combined systematic analysis of nucleotide sequences and morphological characters, and either nucleotide sequence characters or morphological characters alone, resolve these subfamilies at the base of the cupressaceous clade (Gadek et al. 2000; Mao et al. 2012; Rothwell et al. 2011; Yang et al. 2012).

Over the past several years there have been increasingly frequent discoveries and descriptions of basal cupressaceous seed cones in Cretaceous deposits across the Northern Hemisphere (Atkinson et al. 2014a, b; Ghosh et al. 2018; Herrera et al. 2017; LePage 2009; Rothwell and Ohana 2016; Rothwell and Stockey 2018; Stockey et al. 2005, 2018). Together with the systematic relationships resolved for living species (Mao et al. 2012), this increasing sampling density of fossils is beginning to provide empirical evidence for the pattern, mode, and tempo of evolution, and phylogeny within the prominent conifer family Cupressaceae.

The discovery of a virtually complete, anatomically preserved seed cone that conforms to the subfamily Taiwanioideae in late Campanian-early Maastrichtian deposits on the northern Japanese island of Hokkaido further enriches our knowledge of basal Cretaceous Northern Hemisphere conifers during the Late Cretaceous. A new specimen, described herein as

*Mukawastrobus satoi* Stockey, Nishida & Rothwell gen. et sp. nov., further increases the sampling density of extinct cupressaceous conifers. It provides additional information about the morphological diversity and early evolution of one of the most important conifer subfamilies as well as about the mode and tempo of evolution within basal Cupressaceae.

## Materials and methods

The holotype specimen of *Mukawastrobus satoi* was collected from a streambed in the catchment of the Mukawa River, 5 km NE of Mukawa (Hobetsu), Upper Cretaceous Yezo Group by Mr. Ryosuke Sato on the northern Japanese island of Hokkaido. Based upon the most recent stratigraphic work for this area, sediments from which the fossil was collected are most likely part of the Hakobuchi Formation, which is considered to be latest Campanian-Maastrichtian (~ 73–70 Ma) in age (Konishi et al. 2015; Takahashima et al. 2004). This interpretation is also supported by the presence of both the ammonoid, *Neophylloceras hetonaiense* Matsumoto and molluscan fossils of shallow marine origin in the concretion (Shigeta and Nishimura 2013; Shigeta et al. 2017; Toshimitsu et al. 1995). The cone was exposed on the surface of the concretion and cut in longitudinal section. Peels were made from the larger half of the cone (i.e., peel series A), which included both the cone axis in longitudinal section and the cone peduncle in oblique section. The smaller part of the cone was cut in cross section, and serial sections were prepared in that orientation from both the apical and basal segments (i.e., series B top and B bottom). Slides were made using Eukitt xylene-soluble mounting medium (O. Kindler, Freiburg, Germany).

Images were captured with a Better Light digital scanning camera back (Better Light, Placerville, California, USA) using transmitted light, focused through a Leitz Aristophot large-format camera using either Summar lenses or a Zeiss WL compound microscope, and processed using Adobe Photoshop CS5 extended (Adobe Systems Inc., San Jose, California, USA). Three-dimensional reconstructions were rendered by tracing and coloring serial photographs which were then stacked and processed using AMIRA visualization software (TGS Software, San Diego, California, USA).

## Results

### Systematic paleontology

Class Spermatopsida

Order Coniferales sensu Eckenwalder

Family Cupressaceae Gray

Subfamily Taiwanioideae Li

Genus *Mukawastrobus* Stockey, Nishida & Rothwell, gen. nov.

*Generic diagnosis* Conifer seed cones with closely spaced, helically arranged, laminar bract-scale complexes lacking free scale tips; bearing single row of two to three adaxially attached, inverted ovules near bract tip. Cone axis without continuous system of resin canals, bract-scale complexes diverging at ~ 90° in cone midregion and bending distally near apex. Bract-scale complexes with one resin canal originating at base; row of secretory canals in mid-region. Bract trace diverging as single radial bundle, tapering distally, becoming oval before dividing repeatedly at level of seed attachment to produce several terete strands over short distance.

*Etymology* The generic name *Mukawastrobus* refers to the collecting locality on a tributary of the Mukawa River near the town of Mukawa, on Hokkaido, Japan.

**Fig. 1:**
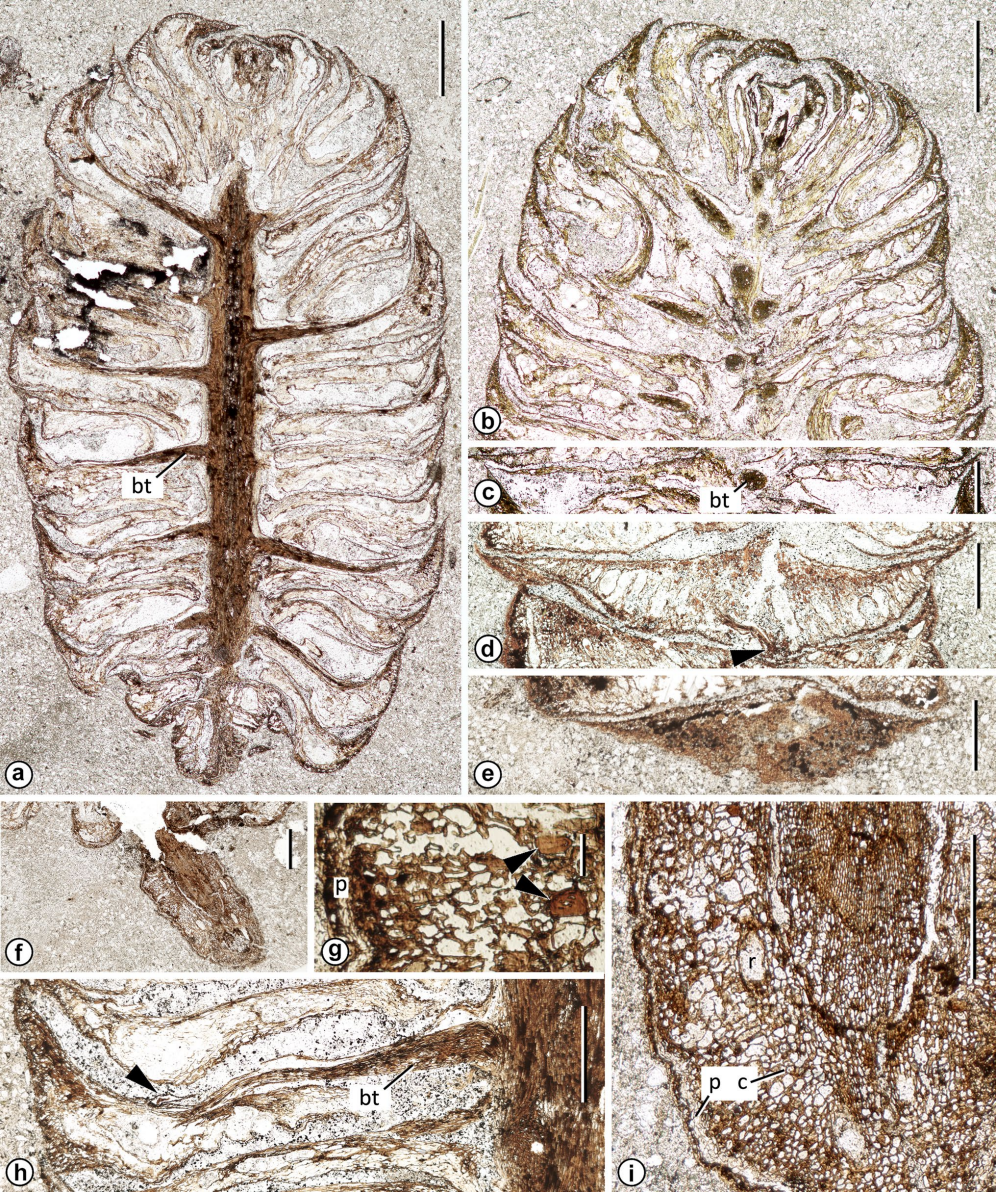
*Mukawastrobus satoi* Stockey, Nishida & Rothwell gen. et sp. nov. (Holotype; NSM-PP9930) **a** Near mid-longitudinal section showing general features of cone. Slide A 31 × 7. Scale bar = 2 cm. **b** Tangential section of apical region showing closely spaced bract/scale complexes with upturned apices, and massive bract traces diverging to complexes. Slide A 55 × 8.5. Scale bar = 2 cm. **c** Cross section of bract/scale near base of complex. Note wide laminar bract with large round trace and row of large resin canals, diverging from axis at nearly 90°. Slide A 55 × 9.5. Scale bar = 1 cm. **d** Cross section of bract/scale complex near apex, at level of distal upturn and where trace (arrowhead) branches. Slide A 101 × 12. Scale bar = 1 cm. **e** Tangential section at cone periphery showing abaxial surface of upturned broad, simple bract-scale complex tip. Slide A 118 × 13. Scale bar = 1 cm. **f** Oblique section of cone peduncle at level of attachment to cone base. Note bent orientation of cone attachment. Slide A 11 × 7.5. Scale bar = 1 cm. **g** Oblique section of peduncle cortex showing parenchymatous ground tissue with sclereids (arrows), somewhat smaller cells with dark contents toward periphery (at left), and several layers of periderm (p) beneath epidermis. Slide A 11 × 80. Scale bar = 0.1 mm. **h** Radial section of cone showing divergence of bract-scale complex with inverted immature ovule (arrowhead) attached to adaxial surface just proximal to level where bract tip bends distally. Note stout bract trace (bt). Slide A 32 × 19. Scale bar = 1 mm. **i** Oblique cross section of peduncle showing continuous cylinder of wood surrounding pith, broad cortex with resin canals (r), narrow zone of periderm (p) beneath epidermis. Slide A 4 × 27. Scale bar = 1 mm

**Fig. 2:**
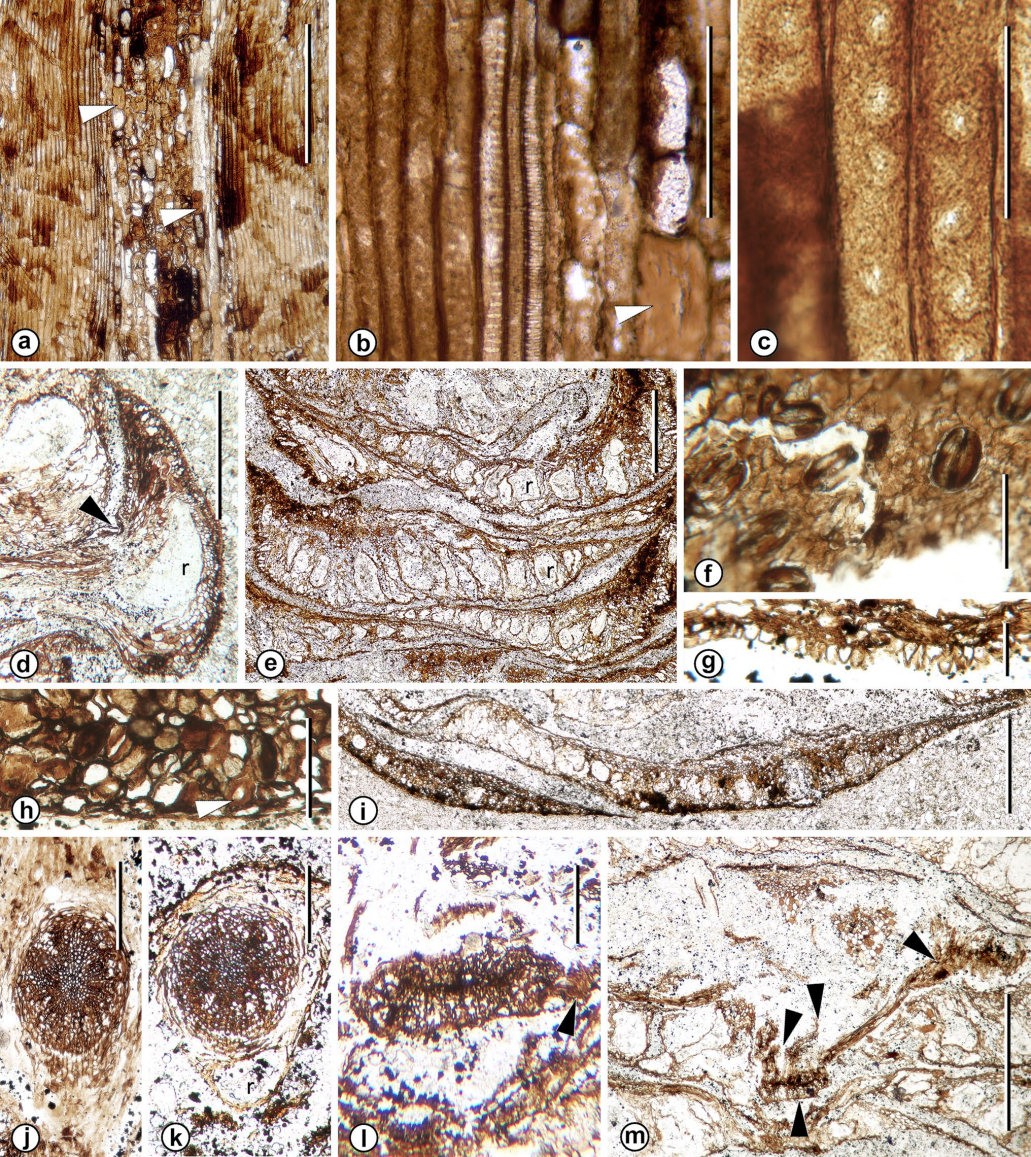
*Mukawastrobus satoi* Stockey, Nishida & Rothwell gen. et sp. nov. (Holotype; NSM-PP9930) **a** Longitudinal section of cone axis showing pith flanked by wood. Note rectangular pith cells, mostly parenchyma, with empty lumens, amber contents or black contents; scattered sclereids (arrowheads). Slide A 22 × 25. Scale bar = 1 mm. **b**. Enlargement of Fig. 2b showing xylem at left, pith cells at right. Arrowhead identifies sclereid. Slide A 22 × 333. Scale bar = 0.1 mm. **c** Radial section of wood showing uniseriate circular pits with broad borders on secondary xylem tracheids. Slide A 36 × 690. Scale bar = 50 µm. **d** Longitudinal section of upturned bract tip with position of ovule attachment identified by arrowhead, resin canal (r), and solidly cellular apex. Slide A 23 × 22. Scale bar = 1 mm. **e** Tangential section of cone showing single row of large resin canals (r) at midlevels of bract/scale complexes. Slide A 119 × 15. Scale bar = 1 mm. **f** Stomata in surface view of abaxial epidermis of upturned bract tip, as enlarged from rectangle in Fig. 1e. Slide A 104 × 170. Scale bar = 0.1 mm. **g** Cross section of bract, showing abaxial epidermal papillae in midregion. Slide A 30 × 188. Scale bar = 50 µm. **h** Ground tissue near tip of bract, showing parenchyma with clear, amber, and black contents and isolated sclereid (arrowhead). Slide B 34 × 180. Scale bar = 0.1 mm. **i** Cross section of cone showing cross sections of upturned bracts immediately distal to upturn (center) and near tip (lower left). Slide B 40 × 17. Scale bar = 1 mm. **j** Cross section of bract trace at level of divergence, showing secondary xylem radiating from center of trace. Note lack of abaxial resin canal at this level. Slide A 49 × 16. Scale bar = 1 mm. **k**. Cross section of bract trace immediately distal to level in Fig. 2j, showing abaxial resin canal (r). Slide A 53 × 14. Scale bar = 1 mm. **l**. Cross section of bract trace immediately proximal to position of ovule attachment and trace division. Note incipiently diverging bundle (arrowhead) at right. Slide A 99 × 26. Scale bar = 0.5 mm. **m**. Cross section of bract at level of upturn and division of trace (upward oriented arrowhead) into several terete strands (downward oriented arrowheads). Slide A 105 × 24. Scale bar = 1 mm

**Fig. 3:**
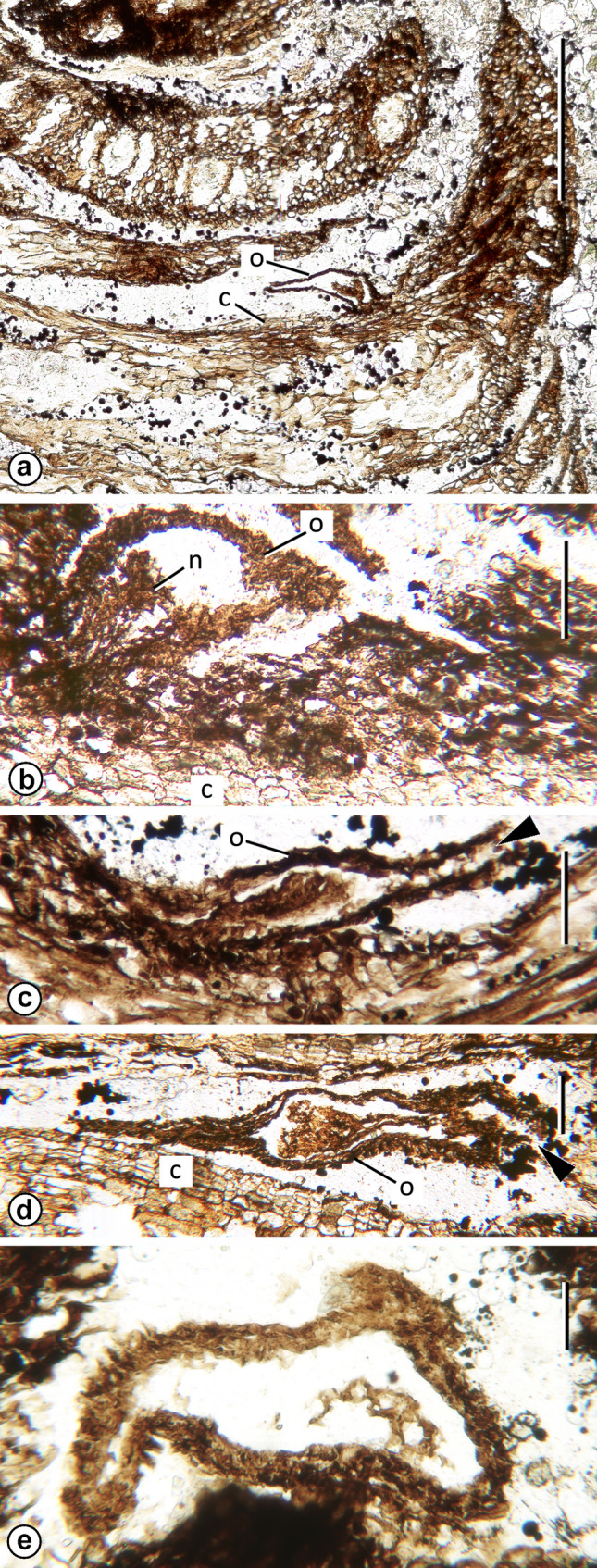
*Mukawastrobus satoi* Stockey, Nishida & Rothwell gen. et sp. nov. (Holotype; NSM-PP9930) **a** Radial section of cone showing immature inverted ovule (o) attached to adaxial surface of bract/scale complex at level where bract tip bends distally. Note zone of closing tissue (c) below ovule. Slide A 47 × 50. Scale bar = 0.5 mm. **b**. Inverted, immature or abortive ovule attached to bract/scale complex, with integument surrounding solid nucellus (n). Note radial rows of cells in closing tissue (c) below ovule attachment. Slide A 38 × 150. Scale bar = 100 µm. **c**. Inverted ovule (o) attached to adaxial surface of bract/scale complex with micropyle (arrowhead) toward right. Note bract/scale complex tip at left, and immature integument surrounding solid nucellus (n). Slide A 47 × 145. Scale bar = 100 µm. **d**. Oblique section of immature ovule (o) with wing at left and micropyle (arrowhead) at right. Note closing tissue (c) adjacent to ovule. Slide A 58 × 90. Scale bar = 100 µm. **e**. Cross section of immature ovule showing wing tissue at upper right. Slide A 36 × 105. Scale bar = 100 µm

**Fig. 4:**
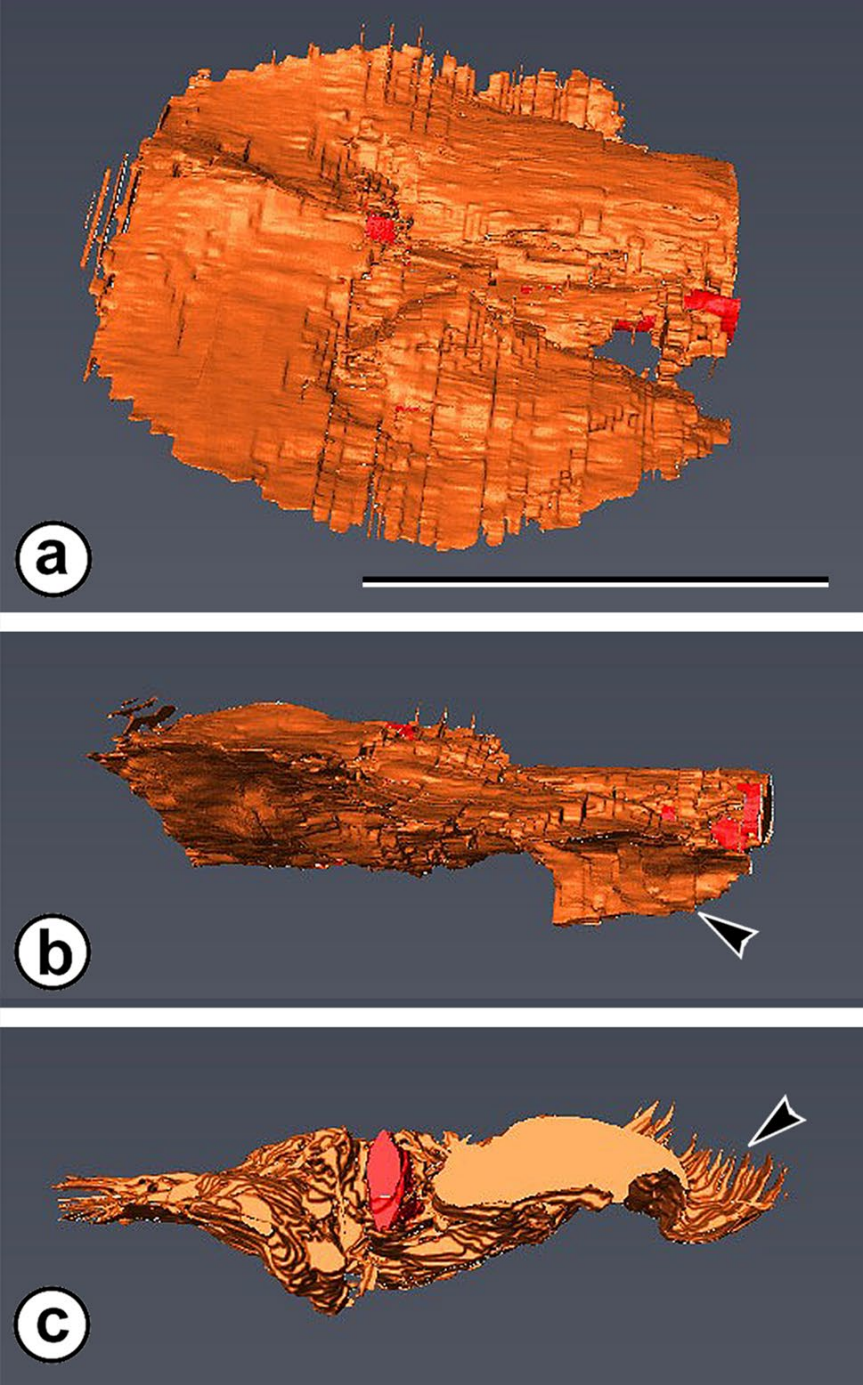
Reconstructions of the bract/scale complex of *Mukawastrobus satoi* gen. et sp. nov. based on stacked peels processed with AMIRA software. All figures ×10. Scale bar = 5 mm. **a** Near top view of scale showing cordate-orbiculate shape, and wide lateral flanges of complex with raised central zone. **b** Side view of scale showing downward dipping edges of bract (arrowhead). Arrow identifies upturned bract tip. **c** View of complex from the axis showing red central xylem strand and downward dipping edge of bract (arrowhead). Note, apparently serrated margin of complex results from distances between adjacent sections and is not a natural feature of the complexes

*Type species Mukawastrobus satoi* Stockey, Nishida & Rothwell sp. nov. (Figs. 1, 2, 3, 4).

*Specific diagnosis* Cone cylindrical, 2.0 cm long, 1.1 cm in maximum diameter. Bract/scale complexes diverging at ~ 90° in mid-region, up to 7 mm long and 8 mm wide; cordate-orbiculate, maximum width immediately distal to divergence from axis. Axial resin canals absent. Single resin canal originating at divergence of bract trace; resin canals increasing in number to ca. 15 in mid-region, diminishing in size and number near distal tip. Bract ground tissue parenchymatous, with scattered sclereids; abaxial epidermis papillate near cone periphery; stomata randomly oriented on abaxial surface of upturned bract tip.

*Etymology* The specific epithet is proposed in recognition of Mr. Ryosuke Sato, Hokkaido, Japan, who collected the holotype specimen and generously made it available for study.

*Holotype* Specimen number NSM-PP9930, is housed in the National Museum of Nature and Science, Tokyo, Japan (Figs. 1–4).

*Type locality* Streambed of tributary of Mukawa (Mu River) near Mukawa (Hobetsu) on the northern Japanese Island of Hokkaido (Lat. 42°59′18.79 ″N; Long. 142°03′10.81 ″E).

*Stratigraphy and age* Late Cretaceous (late Campanian—early Maastrichtian—~ 73–70 Ma).

### Description

*Mukawastrobus satoi* gen. et sp. nov. is represented by a single cylindrical seed cone 2.0 cm long, and 1.1 cm wide (Fig. 1a), that is subtended by a short segment of bent peduncle lacking evidence of subtending leaves (Fig. 1f). The cone has closely spaced, helically arranged bract/scale complexes (Fig. 1a, b) that arise at right angles from the axis in the cone mid-region (Fig. 1a, c, h), and that bend distally at the cone periphery (Figs. 1a, b, h, 2d).

The cone peduncle is 2 mm wide with a smooth outer margin and no evidence of leaves (Fig. 1f, i). It has an angular pith surrounded by a broad zone of secondary vascular tissue, and a largely parenchymatous cortex with resin canals and scattered sclereids (Fig. 1i). Cortical parenchyma cells are isodiameteric with clear lumens except toward the periphery, where there are bundles of cells that are smaller and show dark contents (Fig. 1i). There is a narrow zone of three to four layers of radially aligned cells that form a periderm near the periphery of the peduncle (Fig. 1g, i, at “p”). The outer margin of the peduncle is marked by an epidermis of inconspicuous cells that have light-colored internal contents (Fig. 1i).

The cone axis (Fig. 1a, f, h) is ca. 1.5 mm wide and is similar to the peduncle, showing a central pith constructed of parenchymatous cells with thick walls and either transparent, amber, or dark internal contents (Fig. 2a), and with scattered angular sclereids (Fig. 2a, b, at arrowheads). Primary xylem tracheids with helical to scalariform secondary wall thickenings are preserved adjacent to the pith (Fig. 2b), grading through reticulate pitting (Fig. 1b, at center) to uniseriate circular-bordered pits with round apertures and broad borders (Fig. 2c). The cortex of the cone axis consists of a narrow zone of thin-walled cells (Fig. 1a, h) that are incompletely preserved, and lacks a continuous axial resin canal system.

Bract/scale complexes consist of a large bract with no evidence of a free ovuliferous scale tip (Figs. 1a, h, 2d, 3a, 4a-c). Complexes are up to 7 mm long and 8 mm wide. They diverge from the axis at right angles in the mid-region of the cone, diverge more basally in the proximal region, and become increasingly more apically oriented toward the cone apex (Fig. 1a, b). Individual complexes are flattened and laminar (Figs. 1c, 2e, 4a), extend away from the axis, and then show a nearly 90° distal bend at the cone periphery (Figs. 1a, b, h, 2d, 4b). Each complex broadens immediately as it diverges, reaches its full width only a few mm from the axis (Fig. 4a), has a bluntly pointed apex (Fig. 1e) and a shallowly cordate base (Figs. 1c, 4a). Lateral margins at the base of the complexes often are reflexed toward the base of the cone (Fig. 4b at arrowhead), having a curled appearance in section views (Fig. 1b, at lower right). Ground tissue is largely parenchymatous and inconspicuous as compared to the resin canals of the complexes (Figs. 1a–d, 2e) except in the upturned apical region (Fig. 2d, i), where it is constructed of compact parenchyma with scattered sclereids (Fig. 2h, at arrowhead). Over much of the complex the epidermis is inconspicuous, but at the level where the complex bends distally, abaxial epidermal cells form pointed papillae (Fig. 2g); and on the distal abaxial surface, that is exposed at the cone periphery, there are numerous randomly oriented stomata with conspicuous guard cells (Fig. 2f). The guard cells form a complex that is oval, 45–65 µm long and 35–45 µm wide. Subsidiary cells are not clearly differentiated, but some complexes appear to be weakly cyclocytic (Fig. 2f).

No resin canals accompany the bract trace as it diverges from the cone axis (Fig. 1j), but immediately distal to divergence a single resin canal originates abaxial to the trace (Fig. 1k). The number of resin canals increases rapidly up to ca. 15 (Figs. 1c, d, 2e), with each apparently originating de novo. Resin canals are located on the abaxial side of the bract trace (Fig. 1h), are large and highly conspicuous, and form a single row (Figs. 1c, d, 2e). Distal to the level where the complex bends toward the cone apex the resin canal number and size diminish rapidly (Fig. 2d, i), such that the tip consists largely of compact ground tissue (Fig. 2d, i, at bottom left).

Vascular traces to the cone-scale complex arise as a rod, 1.4–1.6 mm in diameter (Fig. 4c). At that level each consists of rows of tracheids that radiate from the center (Fig. 2j, k). Traces diminish in size, remain undivided, and taper as they extend away from the cone axis (Fig. 1a–c, h). More distally each becomes a laterally oriented oval (Fig. 2l). At the level of seed attachment and distal bend, the bundle divides laterally (Fig. 2l, at arrowhead) over an extremely short distance (Fig. 2m) to form several terete strands that extend toward the bract apex (Fig. 2m). Although the vascular system appears to be located near the abaxial surface of the bract/scale complex in tangential sections of the cone at this level (Fig. 2m), that appearance is due to the acute bend near the bract/scale complex tip. Such bundles actually occur toward the adaxial surface of the bract/scale complex tip.

Seeds are attached to the adaxial surface of bract/scale complex at the level of the abrupt upturn of this complex near the cone periphery (Figs. 1h, 2d, at arrowhead; 3a–c). Only one occurs in any of the available sections, but careful examination of serial sections reveals that two are attached to some complexes and three to others. All seeds are small (< 1 mm long) and inverted with the micropyle oriented toward the cone axis (Figs. 1h, 2d, 3a–c), have a lateral wings, and appear to have been immature or aborted at the time of fossilization (Fig. 3d–e). Individual ovules have an undifferentiated integument of several cell layers (Fig. 3b–e), and a solid nucellus (Fig. 3a–d). Each is oriented with the micropyle facing the cone axis (Figs. 1h, 3a–c) and shows an abscission layer of radially aligned closing cells in the bract tissue immediately adjacent to the position of seed attachment (Fig. 3a–d).

## Discussion

*Mukawastrobus satoi* is a compact conifer seed cone (Fig. 1a), consisting of an axis bearing numerous, imbricating, helically-arranged bract/scale complexes (Fig. 1b) in which the complexes appear to be a laminar bract (Figs. 1a–c, h, 2e) with no free scale tip (Figs. 1a, h, 2d, 3a). Two to three inverted seeds are attached to the adaxial surface near the apex of each complex at the level where the tip bends toward the apex of the cone (Figs. 1h, 2d, 3a). Bract/scale complexes are vascularized by a single, radial bundle (Figs. 2j, k, 4c) that tapers toward the apex of the complex (Fig. 1a, h), and then becomes a laterally expanded oval (Fig. 2i) before dividing.

This overall cone structure and vascularization are most similar to those of the early diverging crown group Cupressaceae, the grade of conifers that at one time formed the family Taxodiaceae Pilger. Distinguishing characters of plants that made up this pre-cladistic concept include relatively large cones with numerous bract-scale complexes and (typically) helical arrangement of complexes. Living representatives of this grade are now recognized as Cupressaceae, Subfamilies Cunninghamioideae (Zucc. ex Endl.) Quinn, Taiwanioideae L.C. Li, Athrotaxoideae L.C. Li, Sequoioideae Saxton, and Taxodioideae Endl. ex K. Koch (Farjon 2005).

Resolution of the relationships among these early diverging lineages of crown group Cupressaceae are not fully resolved (c.f., Farjon 2005; Leslie et al. 2018; Mao et al. 2012), but systematics of living cupressaceous species most often conforms to (Cunninghamioideae + (Taiwanioideae + (Athrotaxoideae + (Sequoioideae + (Taxodioideae + (Callitroideae + Cupressoideae))))); Leslie et al. 2012, 2018; Mao et al. 2012). Among the early diverging lineages, *Mukawastrobus satoi* shares laminar bract-scale complexes with members of Cunninghamioideae, Taiwanioideae, and Athrotaxoideae, while seed cones of Sequoioideae and Taxodioideae have bract scale complexes that are much thicker and peltate at maturity (Farjon 2005; LePage 2009). Species of Cunninghamioideae and Athrotaxoideae have a free scale tip, but in Taiwanoideae and *M. satoi* there is no free scale tip.

Probably because of the high level of extinction among species of early diverging Cupressaceae, living species are easily segregated into the recognized subfamilies (Farjon 2005). This also is true for most extinct species, but a few with intermediate structural features also have been described. For example, Upper Cretaceous cones of *Parataiwania nihongii* and *Mikasastrobus hokkaidoensis* have a relatively large number of helically-arranged, laminar bract/scale complexes with inverted, winged seeds on the adaxial surface, and were originally described as taiwanioid species despite each having a small free scale tip (Nishida et al. 1992; Saiki and Kimura 1993). This latter feature is suggestive of cupressaceous cones of Subfamily Cunninghamioideae, thus weakening their assignment to Taiwanioideae. Nevertheless, we have chosen to continue including *P. nihongii* and *M. hokkaidoensis* in discussions of Subfamily Taiwanoideae until the sampling density of anatomically preserved fossil cupressaceous seed cones is dense enough to more precisely evaluate systematic relationships using cladistic methodology.

### Similarities of *Mukawastrobus satoi* to living *Taiwania cryptomerioides*

The overall features of *M. satoi* are reminiscent of seed cones of the living *Taiwania cryptomerioides*, but *M. satoi* has two to three seeds/scale, rather than the two that characterize *T. cryptomerioides* (Table 1; Farjon 2005; Liu and Su 1983). In *M. satoi* the bract trace divides several times in a short distance near the cone periphery; whereas, in *Taiwania* trace divisions occur over about ½ the length of the bract. The fossil also has an abruptly upturned bract tip (Figs. 1a, b, h, 2d, 3a, 4b at left), and a cone peduncle that lacks leaves (Fig. 1f, i; Table 1). By contrast, in species of *Taiwania* the bract/scale complexes arch gently toward the cone apex, and the peduncle bears dense scale leaves (Farjon 2005; LePage 2009; Liu and Su 1983; Table 1). Like, *Taiwania cryptomerioides, Mukawastrobus satoi* has a single row of resin canals at the midlevel of the laminar bract/scale complex and lacks an axial resin canal system (Table 1). Resin canals of *M. satoi* are larger and more conspicuous (Figs. 1c, d, 2e), however, than in the living *Taiwania cryptomerioides* (see figures of 19 and 20 of Liu and Su 1983).

**Table 1:** Living and anatomically preserved fossil taiwanioid seed cones

Taxon character	*Mukawastrobus satoi gen. et* sp. nov.	*Parataiwania nihongii*	*Mikasastrobus hokkaidoensis*	*Comoxostrobus rossii*	*Stutzeliastrobus foliatus*	*Taiwania cryptomerioides*
Geographic distribution	Hokkaido, Japan	Hokkaido, Japan	Hokkaido, Japan	Vancouver Island, B.C., Canada	Mongolia	Northern Hemisphere
Stratigraphy	Upper Yezo Group	Upper Yezo Group	Upper Yezo Group	Comox Formation, Nanaimo Group	Tevshiin Govi Formation	Cretaceous—recent^a^
Age	Late Cretaceous (late Campanian-early Maastrichtian)	Late Cretaceous (Coniacian-Santonian)	Late Cretaceous (Coniacian-Santonian)	Late Cretaceous (Coniacian)	Early Cretaceous (Aptian-Albian)	Late Cretaceous—recent
Cone attachment	Terminal, bent	Terminal?	Terminal	Terminal?	Terminal, bent	Terminal, bent
Subtending leaves	**Absent**	?	Needle-like	?	Needle-like	Needle-like
Cone shape	Cylindrical	Ellipsoidal	Ellipsoidal	Cylindrical	Obovate—cylindrical	Ellipsoidal
Cone length (mm)	20	22	30–40	> 23	7–32	8–25
Cone width (mm)	11	16	20–35	> 10	6–15	3.9–17
BSC complex arrangement	Helical	Helical	Helical	Helical	Helical	Helical
Angle of BSC divergence at cone center	90°	40–50°	40–50°	~ 10°	90°	40–50°
BSC shape	**Cordate-orbiculate**	Deltoid	Spatulate	Spatulate	Spatulate	Deltoid
BSC base	**Broad**	Narrow	Narrow and stalked	Narrow	Narrow and stalked	Narrow
BSC tip	Convex, pointed	Convex, pointed?	Convex, pointed	Convex	Convex, pointed	Convex, pointed
BSC curvature	**Straight**	Gently adaxial	Gently adaxial	Straight-gently adaxial	Gently adaxial	Gently adaxial
Upturned b/s apex	**Present**	Absent	Absent	Absent	Absent	Absent
Free ovuliferous scale tip	Absent	Present	Present	Absent	Absent	Absent
Axial resin canal system	Absent	Absent?	Present	Absent	?	Absent
Resin canal number	~ 15	> 13	10–14	~ 9	1	5–7
Branching of vascular bundles in BSC	Laterally and repeatedly at level of seed attachment	Primarily laterally and repeatedly over ~ 1/2 of bract length	Laterally and repeatedly over ~ 1/2 of bract length	Laterally in basal ~ 1/2 of bract, unknown distally	?	Laterally and repeatedly over ~ 1/2 of bract length
Stomata	Abaxial on upturned bract tip	?	?	?	2 abaxial patches near bract base	?
Seed attachment on BSC	Near apex	Near apex	Near apex	Midregion	Midregion	Near apex
Seed number/BSC	2–3	4	4–5	6	2–4	2
Seed orientation	Inverted	Inverted	Inverted	Inverted	Inverted	Inverted
Seed wing(s)	Lateral, narrow	Lateral, narrow	Lateral, narrow	Lateral, broad	Distal	Lateral, narrow

Unique characters of *M. satoi* highlighted in bold type

Data from Liu and Su (1983), Nishida et al. (1992), Saiki and Kimura (1993), Ohsawa (1994), Farjon (2005), LePage (2009), Herrera et al. (2017), Stockey et al. (2020)

*BSC* Bract Scale Complex

^a^As defined by LePage (2009) from fossils that are preserved as coalified compressions

Vascularization of bract/scale complexes in *Mukawastrobus satoi* and *Taiwania cryptomerioides* originates as a concentric bundle with several rows of secondary xylem tracheids radiating from the center (c.f. Figure 2j and fig. 20-1 of Liu and Su 1983). Both also divide several times to produce several bundles that are located near the adaxial surface of the complex. However, the divisions in *T. cryptomerioides* occur over a distance of more than half the length of the complex and form a single row at the midlevel (figure 19 of Liu and Su 1983); whereas those of *Mukawastrobus satoi,* all occur within a very short distance at the level where the bract/scale complex bends sharply toward the apex of the cone (Figs. 1d, 2m). Nowhere along the length of the horizontal part of the bract/scale in *M. satoi* does a row of bundles appear in cross sections of the complexes (Fig. 2e). Like those of *Taiwania cryptomerioides*, the bundles in the upturned region of the bract/scale complexes in *Mukawastrobus satoi* do form a single row, but they are smaller at that level (Fig. 2m) and difficult to identify in cross sections near the tip (Fig. 2i). All of the seeds that remain attached to the bract/scale complexes of *M. satoi* are either very immature or abortive (Figs. 1h, 2d, 3a–e). This is consistent with senescent cones of the living *Taiwania cryptomerioides*, where a large percentage of the seeds are abortive, and remain in the cones after fertile seeds have been shed (Buchholz ex. Schmid 2013).

### Similarities of *Mukastrobus satoi* to other fossil taiwanioid seed cones

Taiwanioid seed cones have been described from throughout the Northern Hemisphere from the Lower Cretaceous (Albian) through the Pleiocene, with at least twelve species of six genera having been described to date (Table 1). These include seven species of *Taiwania* that occur as compression fossils (LePage 2009), four species of anatomically preserved cones that are assigned to extinct monotypic genera (i.e., *Parataiwania nihongii* M. Nishida, Ohsawa et H. Nishida, *Mikasastrobus hokkaidoensis* Sakai et Kimura (Saiki and Kimura 1993; Nishida et al. 1992), *Comoxostrobus rossii* Stockey, Rothwell et Atkinson 2020, and *Mukawastrobus satoi* described here), and one species based on lignified specimens (i.e., *Stutzeliastrobus foliatus* Herrera, Shi, Knopf, Leslie, Ichinnorov, Takahashi, Crane et Herendeen 2017). The compressed fossil cones all tend to be ellipsoidal like those of *T. cryptomerioides,* and they fall almost completely within the range of variation for length and maximum diameter that characterizes the living species (Farjon 2005; LePage 2009; Liu and Su 1983).

*Stutzeliastrobus foliatus* Herrera, Shi, Knopf, Leslie, Ichinnorov, Takahashi, Crane and Herendeen, from the Early Cretaceous of Mongolia, is a lignified Early Cretaceous conifer represented by leafy branching shoots with terminal seed cones that conform to features of taiwanioid Cupressaceae. Although not assigned to the Taiwanioideae by Herrera et al. (2017) the compact seed cones with imbricating laminar bract/scale complexes of this species have no free scale tip, and *S. foliatus* occurs at an adjacent node to *Taiwania cryptomerioides* on the stem of the tree in results of their phylogenetic analysis (Herrera et al. 2017). *Stutzeliastrobus foliatus* is among the most ancient taiwanioid conifers and is unique because the mode of preservation provides evidence for both external morphology and cuticular features, as well as some cellular anatomy of internal tissues, combining the two types of preservation (Table 1).

Specimens of *S. foliatus* have needle leaves with two bands of abaxial stomata, and terminal cones attached to a bent, leafy peduncle. Therefore, attachment is much like that of *Mukawastrobus satoi*, except that the peduncle of *M. satoi* is not leafy. The holotype of *M. satoi* falls well within the range of size variation for seed cones of *S. foliatus*, and cones of the latter species range in shape to nearly cylindrical, like those of *M. satoi* (c.f., Figure 1a to figure 4 of Herrera et al. 2017). Both taxa have imbricating laminar bract/scale complexes with a pointed tip, but those of *S. foliatus* are narrowly attached and stalked, while those of *M. satoi* have a cordate base. There are up to 15 resin canals in the bract/scale complexes of *M. satoi*, while *S. foliatus* has only a single, centrally located resin canal. Stomata are located abaxially on the upturned bract tip of *M. satoi*; whereas, those of *S. foliatus* occur in two abaxial bands near the base of the abaxial surface. *Mukawastrobus satoi* bears 2–3 adaxial seeds with lateral wings near the tip of the bract/scale complex, while in *S. foliatus* 2–4 adaxial seeds with a proximal wing are attached in the midregion of the bract/scale complex. In both species the seeds are inverted.

*Parataiwania nihongii* was also described from a calcium carbonate nodule from the Upper Yezo Group, Late Cretaceous of Hokkaido, Japan (Nishida et al. 1992). This ellipsoidal cone comes from a Coniacian-Santonian locality exposed in the riverbed of the Kumaoizawa River near the upper reaches of Lake Katsurazawa, Ikushumbetsu, Mikasa City, making this taxon older than *Mukawastrobus. Parataiwania nihongii* has bract/scale complexes that are composed mostly of bract, but that have a very small free ovuliferous scale tip (Table 1). Bract/scale complexes in *Parataiwania* arise at an angle of 40°–50°, while those of *Mukawastrobus* arise at nearly right angles to the cone axis. There are four seeds/scale, unlike the two to three reported in *Mukawastrobus,* each with two lateral wings (Table 1). The bract/scale vascular strand in *Parataiwania* arises as a single wedge-shaped bundle that becomes adaxially convex and expands rapidly into an elongate band in the scale. More distally it divides into three strands, and then up to 13 traces about mid-scale (Nishida et al. 1992). This branching pattern of the vasculature is unlike the closely spaced branching near the scale tip in *Mukawastrobus.* There is a single resin canal abaxial to the vascular strand of the bract/scale complex as in *Mukawastrobus*, however, it arises from an axial system in *Parataiwania*, and two canals arise de novo in the scale. Up to 13 resin canals, which remain abaxial to the vasculature, have been observed in distal bract/scale sections in *Parataiwania* (Nishida et al. 1992). These canals have been shown to have an epithelium of two to three cells and copious resinous contents, while in *Mukawastrobus* and *Taiwania* (Liu and Su 1983) there is a single-celled epithelium.

*Mikasastrobus hokkaidoensis* cones, also from the Upper Yezo Group, Late Cretaceous of Hokkaido, come from Coniacian-Santonian localities exposed around the Kumaoizawa and Yubarigoezawa valleys making this taxon also older than *Mukawastrobus* (Saiki and Kimura 1993). Like those of *Taiwania* and *Stuzeliastrobus, Mikasastrobus* cones are borne terminally on leafy shoots (Saiki and Kimura 1993; Table 1). *Mikasastrobus* cones are larger than the other taxa described above and bract/scale complexes are borne at angles of 40°–50°, unlike those of *Mukawastrobus* that are borne at nearly right angles to the cone axis. Unlike the cone axis of *Mukawastrobus, Mikasastrobus* has an extensive system of axial resin canals, the central one of which enters the bract/scale complex abaxial to the vascular trace. At more distal levels, up to 10–14 canals appear in the bract/scale complex in *Mikasastrobus* (Saiki and Kimura 1993). *Mikasastrobus* cones bear four to five seeds per bract/scale complex (Table 1), unlike the two to three seeds/scale seen in *Mukawastrobus*. Unlike those of *Mukawastrobus* or the other taxa, seeds of *Mikasastrobus* were borne on short parenchymatous pads (Saiki and Kimura 1993).

Recently, *Comoxostrobus rossii* Stockey, Rothwell and Atkinson was described based on a seed cone from the Late Cretaceous (Coniacian) of Vancouver Island, Canada (Stockey et al. 2020), making this taxon older than *Mukawastrobus.* The cone apex and base were not preserved and the mode of attachment is not known for *Comoxostrobus* (Stockey et al. 2020), but the general shape is cylindrical as in *Mukawastrobus* (Table 1). Bract/scale complexes were borne at angles of about 10°, making the cone rather compact compared to Mukawastrobus where bract/scale complexes are borne at 90° angles (Table 1). Bract/scale complexes are spatulate, narrower at the base, and lack an upturned tip (Stockey et al. 2020; Table 1). Like *Mukawastrobus*, and *Taiwania*, *Comoxostrobus* appears to lack an axial resin canal system (Table 1). There is a single, larger, central resin canal in *Comoxostrobus* as in *Mukawastrobus*, but there are fewer lateral resin canals (only about eight) in *Comoxostrobus* than in *Mukawastrobus* where larger numbers are typical (Table 1). The vascular system of *Comoxostrobus*, like that of *Taiwania*, and *Mukawastrobus* arises as a single circular strand, but divides into two and then four vascular strands, unlike the repeated branching seen in *Mukawastrobus* and other taiwanioids (Table 1). Most of the branching of the vascular system in *Comoxostrobus* takes place near the base of the bract/scale complex rather than near the distal tip as in *Mukawastrobus* (Table 1). There are six large seeds per scale with lateral, fleshy wings in *Comoxostrobus*, and unlike those seen in *Mukawastrobu*s, these are all fully developed with megagametophytes and embryos (Stockey et al. 2020). Seed wings in *Comoxostrobus* are very similar to those reported in *Taiwania* and the wings overlap each other (Liu and Su 1983), but they are borne in three rows of two unlike the single row of two seen in *Taiwania*, and the two to three seeds in *Mukawastrobus* in which they are borne nearer the scale apex.

The similarity of general structure of compressed specimens to the living species of *Taiwania* throughout the Cretaceous, Paleogene, and Neogene has prompted LePage (2009) to propose that there has been remarkable evolutionary stasis among Northern Hemisphere taiwanioids since the Early Cretaceous. By contrast to the compressed taiwanioid seed cones, and in agreement with *M. satoi*, the previously described anatomically preserved fossil taiwanioid seed cones and *S. foliatus* are clearly distinct from species of *Taiwania* (Table 1). All, except *S. foliatus*, and *C. rossii* are from the Late Cretaceous (Upper Yezo Group) of central Hokkaido, Japan. Whereas, *Parataiwania nihongii* and *Mikasastrobus hokkaidoensis* occur in Coniacian-Santonian deposits, *M. satoi* is from a somewhat younger (i.e., late Campanian-early Maastrichtian) source. *Stutzeliastrobus foliatus* is from central Asia (Mongolia) and considerably older than the other three (i.e., Lower Cretaceous, Aptian-Albian). These five monotypic genera are distinguished from species of *Taiwania* and from each other by relatively subtle seed cone characters that would be difficult to observe from compression fossils (Table 1). Pertinent distinguishing characters of the four fossil taxa include general bract/scale complex shape and divergence angle, presence or absence of an upturned apex, presence or absence of a free scale tip, number, orientation and attachment position of seeds, nature of the seed wing, features of vascularization and resin canals, organ histology, and stomatal distribution (Table 1).

While cones of *Mikasastrobus* are somewhat larger than those of the other four genera and those of *Mukawastrobus* are somewhat more cylindrical than most of the others (Table 1), were they preserved as compression fossils all of them probably could not be clearly distinguished from either living *Taiwania* or many of the previously described taiwanioid seed cones from the Cretaceous and Cenozoic fossil record (i.e., see Table 1 of LePage 2009). Within this context, the anatomically preserved fossil taiwanioid cones reveal much more evolutionary diversity among extinct taiwanioids than presently suspected (LePage 2009).

This situation is concordant with evolutionary changes that have occurred within the cupressaceous sequoioid genus *Metasequoia*, where either anatomical preservation (i.e., *M. millerii* Rothwell et Basinger; Basinger 1981, 1984; Rothwell and Basinger 1979) or an extremely large assemblage of specimens that document ranges of variation for all of the organs of the plant (i.e., *M. foxii* Stockey et al. 2001) is required to distinguish among similar species that are separated from each other by particularly subtle characters (Stockey et al. 2001). As a result, *Mukawastrobus satoi,* and the other anatomically preserved fossil taiwanioid seed cones reveal an evolutionary radiation during the Late Cretaceous, and much wider diversity and more evolutionary change within Cretaceous and Cenozoic taiwanioids than has been recognized previously.
